# Sequence determinants of specific pattern-recognition of bacterial ligands by the NAIP–NLRC4 inflammasome

**DOI:** 10.1038/s41421-018-0018-1

**Published:** 2018-05-08

**Authors:** Jingyi Yang, Yue Zhao, Peng Li, Yi Yang, Ejuan Zhang, Maohua Zhong, Yaoming Li, Dihan Zhou, Yuan Cao, Mengji Lu, Feng Shao, Huimin Yan

**Affiliations:** 1 Mucosal Immunity Research Group, State Key Laboratory of Virology, Chinese Academy of Sciences, Wuhan Institute of Virology, 430071 Wuhan, China; 2 National Institute of Biological Sciences, 102206 Beijing, China; 3 Institute of Virology, University Hospital of Essen, University Duisburg-Essen, Hufelandstrasse 55, 45122 Essen, Germany

## Abstract

The NLR apoptosis inhibitory proteins (NAIPs) function as specific cytosolic receptors for bacterial ligands to form the NAIP–NLRC4 inflammasome for anti-bacterial defenses. In mice, NAIP5/6 and NAIP2 recognize bacteria flagellin and the rod protein of the type III secretion system (T3SS), respectively. However, molecular mechanism for specific ligand pattern-recognition by the NAIPs is largely unknown. Here, through extensive domain swapping and truncation analyses, three structural domains, the pre-BIR, BIR1, and HD1, in NAIP2 and NAIP5 are identified, that are important for specific recognition of their respective ligand(s). The three domains are sufficient to confer the ligand specificity for NAIP2. Asp-18, Arg-108, and Arg-667, respectively, in the pre-BIR, BIR1 and HD1 of NAIP2 are further identified, each of which is essential for efficient binding to the rod protein. To our surprise, we find that the C-terminal leucine-rich repeat domain is dispensable for NAIP2 recognition of the T3SS rod protein, but is required for NAIP5 binding to flagellin. At the ligand side, we discover that the C-terminal 35 residues in flagellin are crucial for binding to NAIP5. Among the 35 residues, three critical residues are identified, which determine flagellin recognition by NAIP5 and subsequent inflammasome activation. The differences in the three amino-acid residues among flagellins from various pathogenic and commensal bacterial species correlate well with whether they are susceptible to NAIP5-mediated immune detection. Taken together, our studies identify critical sequence and amino-acid determinants in both NAIP receptors and the bacterial ligand flagellin that are important for the specificity of the pattern-recognition.

## Introduction

NLRs, the nucleotide-binding domain (NBD), and leucine-rich repeat (LRR)-containing proteins, exhibit versatile functions in innate and adaptive immunity^1^. The NLR apoptosis inhibitory protein (NAIP) family members are cytosolic pattern-recognition receptors that detect bacterial ligands to form the NAIP–NLRC4 inflammasome for anti-bacterial defenses^2–7^. Upon interaction with bacterial ligand, a single NAIP molecule changes its conformation to interact with and activate NLRC4, which prompts assembly of an oligomeric NAIP–NLRC4 inflammasome^8, 9^. Thereafter, the NAIP–NLRC4 inflammasome activates the cysteine protease caspase-1 to cleave the precursors of proinflammatory cytokines interleukin-1β (IL-1β), IL-18, and gasdermin D^10, 11^, which trigger pyroptotic cell death^12^.

There are seven paralogs of NAIP in mice. Mouse NAIP1 and NAIP2 bind the needle and rod proteins of the type III secretion system (T3SS), whereas NAIP5 and NAIP6 bind cytosolic flagellin^13, 14^. The NAIP–NLRC4 inflammasomes respond to a broad spectrum of pathogens, such as *Salmonella*, *Shigella*, and *Legionella*, in which NAIPs exhibit high ligand specificity^13–16^. The NAIPs are also required for lethal inflammasome activation in a ligand-specific manner. Mouse NAIP paralogs share a high degree of amino-acid identity and the same basic architecture, and all converge to activate NLRC4. Four functional NAIPs (NAIP1, 2, 5, and 6) have been discovered in inbred C57BL/6 mice, whereas additional NAIP4 and NAIP7 found in inbred 129 mice though the functions of NAIP4 and 7 have not been determined yet. In contrast, only a single NAIP is discovered in humans that can detect T3SS needle protein or flagellin via two different splice forms, but hNAIP cannot detect T3SS rod protein^14, 17^. How and why different members of the NAIP are evolved to respond to a diverse set of ligands and agonists is an intriguing but unanswered question^18–20^. The molecular basis for the specific pattern-recognition of divergent bacterial ligands by the NAIP–NLRC4 inflammasome warrants further investigation.

Tenthorey et al.^21^ mapped the NAIP specificity domain for pattern-recognition using a panel of 43 chimeric NAIPs, of which, remarkably, 31 (72%) retained at least some function. They found that the ligand specificity was mediated by the NBD-associated helical region containing helical domain 1 (HD1), winged helix domain (WHD), and helical domain 2 (HD2) located in an internal region of NAIP molecule, and that the annotated NAIP LRR domain was dispensable for ligand specificity. However, the chimera containing the whole NBD-associated helical region of NAIP5 and other regions of NAIP2 in their study was still unable to respond to NAIP5 ligand^21^, suggesting that other structural regions should also be involved in specific ligand binding. At the ligand side, the C-terminal 35 (C35) amino-acids portion of flagellin is essential for the binding capacity of flagellin to NAIP5^22^. More particularly, three conserved leucine residues (3L) located in the most terminal five or six amino-acid residues of the C35, such as L502, L504, and L505 in *Salmonella*, L470, L472, and L473 in *Legionella* flagellin are identified to be critical for binding to NAIP5 and activating the inflammasome^14, 22^. However, we also found that many 3L-containing flagellins such as those from enteropathogenic *Escherichia coli* (EPEC), enterohaemorrhagic *E. coli* (EHEC), *Shigella flexneri*, *Chromobacterium violaceum,* and *Burkholderia thailandensis* could not bind to NAIP5^14^. A 3L-containing flagellin from *E. coli* K12 strain (KF) showed > 10 times less efficient than flagellin from *Salmonella typhi* (SF) in activating NLRC4 pathway, though these two Enterobacteriaceae bacteria are highly related^23^. These suggest some other unidentified amino-acid determinants than the 3L in the C35 are also critical and essential for flagellin binding to NAIP5 and subsequent NLRC4 inflammasome activation.

In the present study, by extensive domain swapping, truncation, and site mutation analyses, we tried to identify sequence and amino-acid determinants in both NAIP receptors and the bacterial ligands, which are critical for specific pattern-recognition of distinct flagellin.

## Results

### Structural regions in NAIP2 critical for specific recognition of the T3SS rod protein

**Fig. 1:**
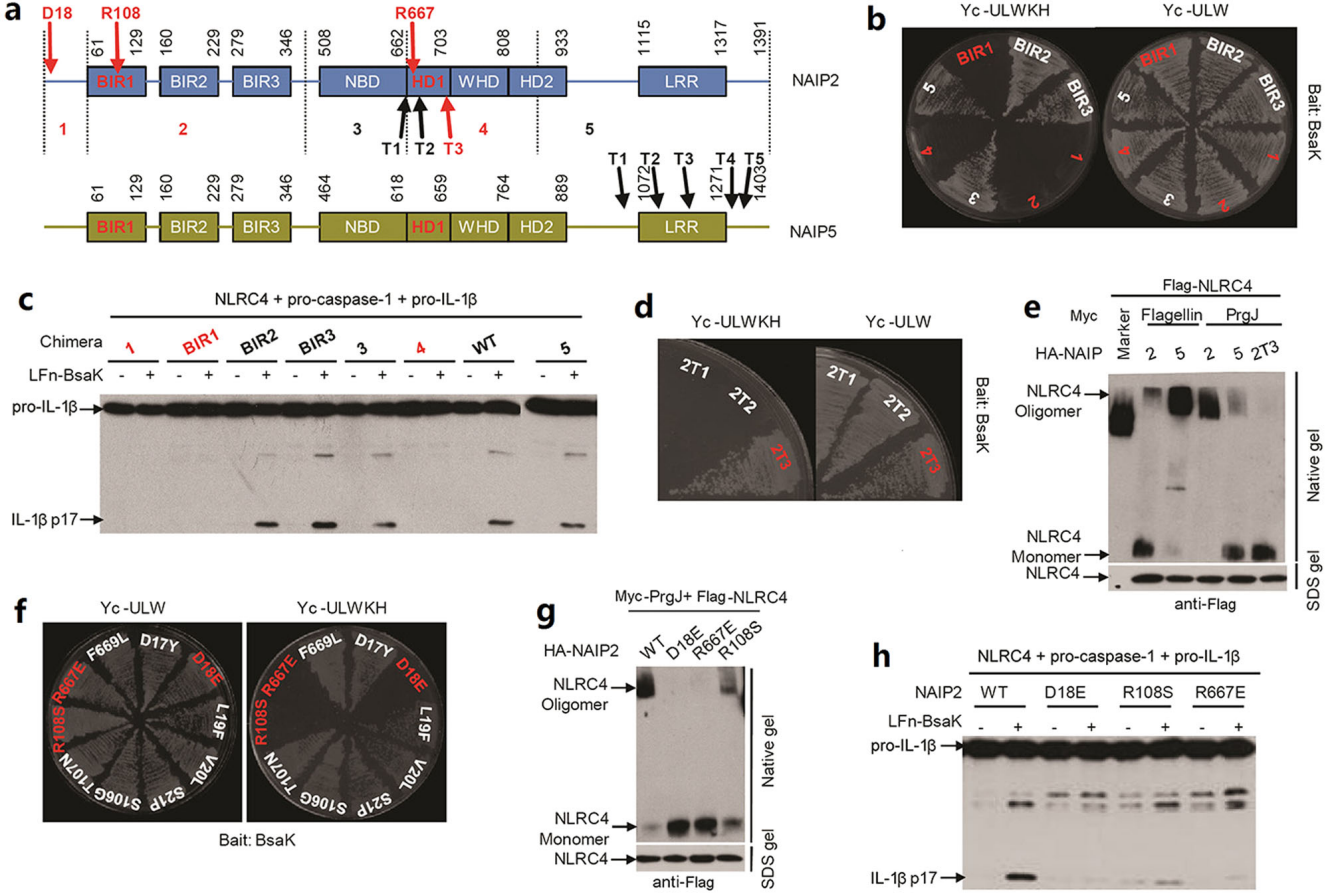
Pre-BIR, BIR1, and HD1 domain of NAIP2 determine the specific binding to its ligand T3SS rod protein. **a** Schematic domain of NAIPs predicted by the NCBI Conserved Domain Database. The NBD, HD1, WHD, and HD2 domains were annotated according to homology with NLRC4. The full-length NAIP protein is divided into fine fragments and illustrated by dash lines. Chimeras were constructed by exchange each fragments of NAIP2 with corresponding ones in NAIP5. The three critical amino acids that determines rod protein binding to NAIP2 are indicated by arrows on the top of the NAIP2 scheme, whereas below are sites for three different truncations. **b** Yeast two-hybrid assays of interactions between *B. thailandensis* rod protein BsaK and NAIP2/5 chimeras in which indicated NAIP2 region is substituted by NAIP5. **c** BsaK induced activation of the NAIP Chimera/NLRC4 inflammasome in reconstituted 293T cells. Lysates from 293T cells transfected with indicated plasmid combinations and stimulated with LFn-BsaK were analyzed for mature IL-1β (p17) by immunoblotting. **d** Yeast two-hybrid assays of interactions between BsaK and NAIP2 truncations indicated in **a**. **e** Blue Native PAGE assay to monitor the formation of the NAIP/NLRC4 inflammasome. 293T cells were transfected with NLRC4 and the indicated NAIPs or their truncation and 6-Myc tagged PrgJ or flagellin. After 36 h, cells were harvest and lysed in Native lysis buffer and subjected to Blue Native PAGE analysis. A fraction of the cell lysates were also subjected to denatured SDS–PAGE for protein expression and loading. **f** Yeast two-hybrid interaction assays of interactions between BsaK and different NAIP2 mutants. **g** Blue Native PAGE assay to test the ability of the NAIP2 mutants in forming inflammasome. **h** BsaK induced activation of the NAIP2 mutant/NLRC4 inflammasome in reconstituted 293T cells. Expression of transfected inflammasome components for **c**, **e**, **g** and **h** is in Supplementary Figure S1

First, we wanted to investigate structural regions in different NAIPs that are responsible for recognizing the cognate bacterial ligands. The NAIPs share high-sequence homology and adopt the same modular structural architecture. According to the relative differential sequence identify among different regions in NAIPs, we divided the full-length protein into five regions, regions 1–5 from the N to the C terminus as illustrated in Fig. 1a, which were subjected to swapping between NAIP2 and NAIP5. In the yeast two-hybrid interaction assay, we found that individual substitution of region 1, 2, and 4, but not region 3 and 5, of NAIP2 with the corresponding part of NAIP5 resulted in largely diminished binding to the NAIP2 ligand, the T3SS inner rod protein BsaK from *B. thailandensis* (Fig. 1b). Region 2 contains the three baculovirus inhibitor of apoptosis repeats (BIRs, BIR1-3). Further substitutions analyses of the three BIRs revealed that BIR1, but not BIR2 and BIR3, was required for determining the specific recognition of BsaK by NAIP2 (Fig. 1b). Consistently, these binding deficient chimeras also showed defects in supporting BsaK activation of the NAIP–NLRC4-caspase-1 inflammasome pathway-mediated IL-1β maturation reconstituted in 293T cells (Fig. 1c and Supplementary Figure S1a). Thus, these data identify three structural regions (the pre-BIR domain, BIR1 and the HD1-WHD domain) in NAIP2 critical for determining specific recognition of the T3SS rod protein.

As the LRR domain (region 5) was not involved in determining specific binding of NAIP2 to BsaK, we constructed a series of progressive truncations of the C-terminal portion of NAIP2 and tested their interaction with BsaK. These analyses identified a NAIP2T3 truncation, containing amino acids from the N terminus to the HD1 domain (without the WHD, HD2, and LRR domains), which was sufficient to interact with BsaK in the yeast two-hybrid system (Fig. 1d), and in 293T co-precipitation system (Supplementary Figure S2 left panel). For enteropathogenic *E. coli* (EPEC) T3SS rod protein EscI, which has much less NAIP2-binding ability, NAIP2T3 was also sufficient to interact with EscI (Supplementary Figure S2 right panel). Native polyacrylamide gel electrophoresis gel (PAGE) analysis revealed that NAIP2T3 was unable to form a high-molecular-weight complex with NLRC4 in response to the T3SS rod protein stimulation (Fig. 1e and Supplementary Figure S1b). Taken together, these analyses demonstrate that the C-terminal region from WHD to LRR domain in NAIP2 is dispensable for binding to the rod protein; no matter the rod protein has high or low binding ability, though the region is essential in forming inflammasome.

To locate the exact residues in NAIP2 that determine specific ligand binding, we carefully examined the sequence differences between NAIP2 and NAIP5 and performed further substitution of every differential sequence regions within the pre-BIR domain, BIR1 and HD1 with the corresponding sequences of NAIP5. These resulted in identification of residues 17–21 within the pre-BIR domain, 106–108 within BIR1 and 667–669 within HD1 that were important for specific binding to the rod protein. In subsequent point mutation analyses, three single-point mutants of NAIP2, D18E, R108S, and R667E, were found to be defective in binding to BsaK in the yeast two-hybrid analyses (Fig. 1f). Furthermore, these three single-point mutants of NAIP2 abolished the oligomerization of NLRC4 in response to the rod protein (PrgJ) stimulation (Fig. 1g and Supplementary Figure S1c), and failed to support the rod protein-induced NAIP2-NLRC4 inflammasome activation reconstituted in 293T cells (Fig. 1h and Supplementary Figure S1d).

### The LRR is required in NAIP5 recognition of flagellin

**Fig. 2:**
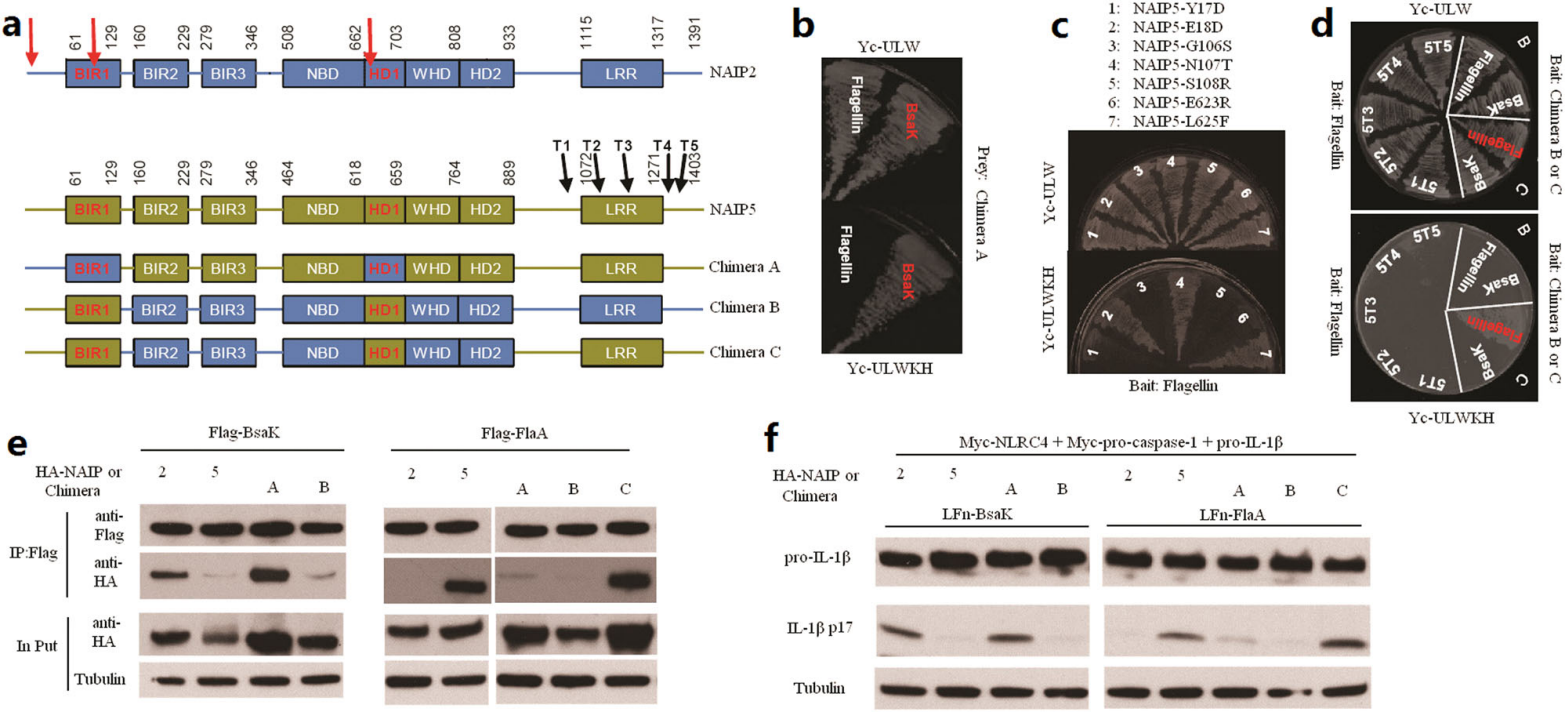
Unlike rod protein, flagellin require LRR domain of NAIP5 for binding. **a** Schematic of predicted NAIP domains in NAIP2, NAIP5, and NAIP2/5 chimeras. The sites of amino acids substitution or truncation are labeled by arrow. **b** Yeast two-hybrid assays of interactions between BsaK or *L. pneumophila* flagellin FlaA and NAIP Chimera A. **c** Yeast two-hybrid interaction assays of interactions between flagellin FlaA and different NAIP5 mutants. **d** Yeast two-hybrid interaction assays of interactions between BsaK or flagellin FlaA and different NAIP chimeras or NAIP5 truncations indicated in **a**. **e** Co-immunoprecipitation assay of interactions between BsaK or flagellins FlaA and NAIP2, NAIP5, or NAIP2/5 chameras in 293T cells. **f** Reconstitution of flagellin FlaA activation of the NAIP chimera/NLRC4 inflammasome in 293T cells. Expression of transfected inflammasome components for **f** is in Supplementary Figure S3

We then turned to examine NAIP5 recognition of flagellin and performed similar sequence and domain substitution analyses on the NAIP5 background (Fig. 2a). For this, three chimera molecules (A, B, and C) as illustrated in Fig. 2a were generated and subjected to yeast two-hybrid interaction assays with both flagellin (FlaA from *Legionella pneumophila*) and BsaK. Chimera A contained pre-BIR, BIR1, and HD1 of NAIP2 with the rest of sequences from NAIP5, and was found be defective in binding to FlaA (Fig. 2b), suggesting that the three regions or at least one of them are critical for flagellin recognition. Importantly, Chimera A could bind to BsaK just like the native NAIP2, suggesting that the three regions are not only required but also sufficient for determining specific recognition of the rod protein ligand (Fig. 2b). We further performed extensive single residue substitution analyses of all differential residues between NAIP5 and NAIP2 in the three structural regions, out of which Y17D, G106S, S108R, and E623R were found to lose the ability to bind to flagellin (Fig. 2c), suggesting that these four residues are critical for NAIP5 recognition of flagellin.

Contrasting to Chimera A, Chimera B possesses the pre-BIR domain, BIR1 and HD1 of NAIP5 and other regions of NAIP2 (Fig. 2a). However, Chimera B showed no binding to flagellin (Fig. 2d), indicating that pre-BIR, BIR1, and HD1 are not sufficient for recognizing flagellin, which is in contrast to the situations in NAIP2. To further investigate NAIP5 recognition of flagellin, we replaced the LRR domain and surrounding regions in Chimera B with the equivalent fragment of NAIP5 (Chimera C). Notably, Chimera C was found to be fully capable of binding to flagellin, but not BsaK (Fig. 2d). We then performed progressive truncation of NAIP5 from the C terminus (T1–T5) (Fig. 2a). NAIP5T1, T2, T3, and T4 all lost the ability to bind to flagellin in the yeast two-hybrid system while the longest truncation NAIP5T5 that contained the entire LRR domain remained certain level of interaction with flagellin (Fig. 2d). These results together suggest that NAIP5, different from NAIP2, requires the LRR domain to bind to its ligand in innate immune recognition.

Consistent with results of the yeast two-hybrid interaction assay, Chimera A but not Chimera B could be co-precipitated by BsaK when both proteins were expressed in 293T cells (Fig. 2e). Although neither Chimera A nor Chimera B showed a co-immunoprecipitation interaction with flagellin, Chimera C was readily precipitated by flagellin from 293T cells (Fig. 2e). Function-wise, Chimera A but not Chimera B could support BsaK stimulation of the NAIP–NLRC4 inflammasome activation to a similar extent as native NAIP2 in the 293T reconstitution system. For flagellin activation of the reconstituted inflammasome, only Chimera C but not Chimera A and Chimera B could substitute NAIP5 and showed full functionality (Fig. 2f and Supplementary Figure S3).

### The C-terminal 35 residues in flagellin are essential for flagellin-NAIP5 interaction

**Fig. 3:**
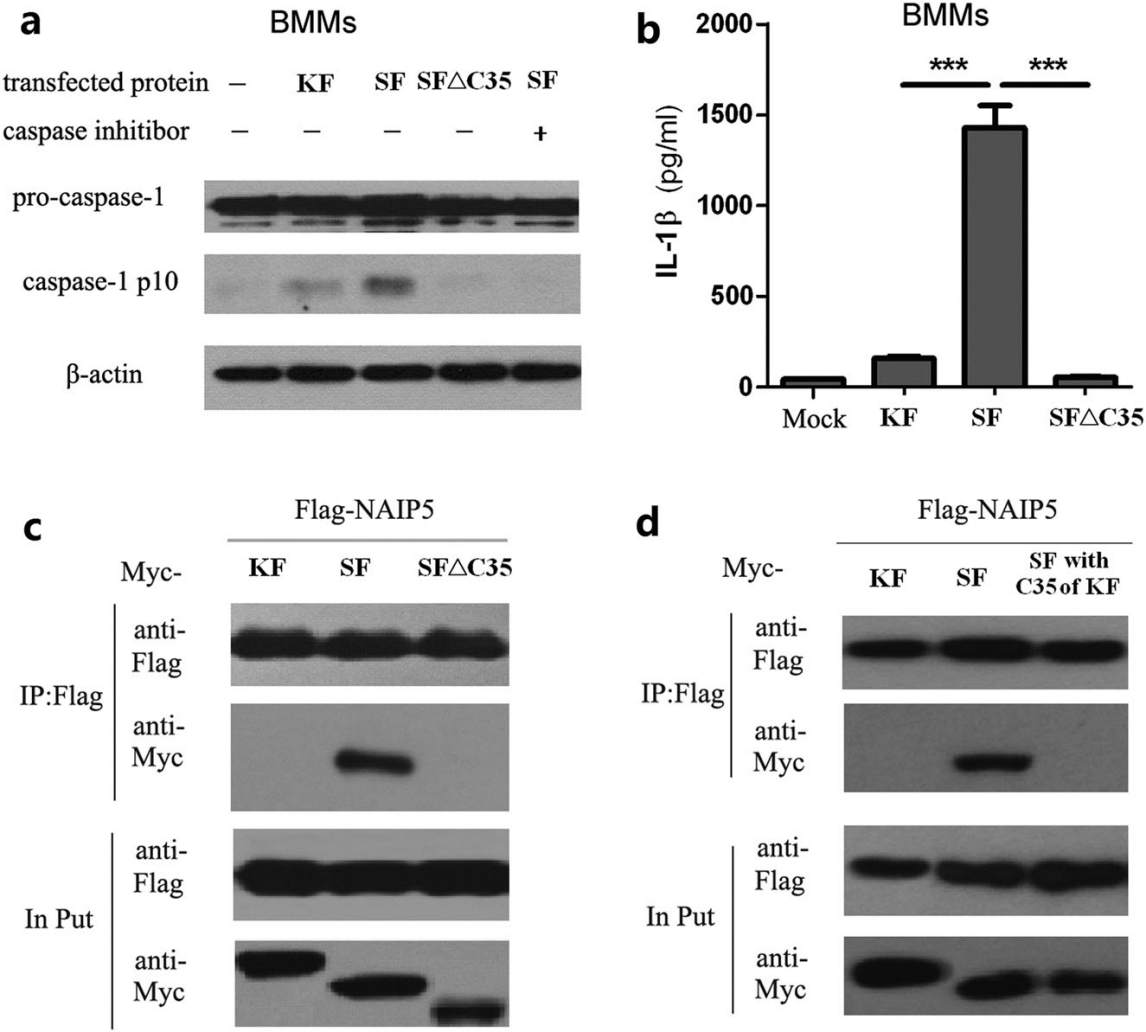
Recombinant mutant flagellin of *Salmonella typhi* (SF) without its C-terminal 35 amino acids fails to activate NLRC4 pathway. **a** Caspase-1 activation detected 1 h after transfection of 100 nM flagellins in LPS pretreated BMMs. **b** IL-1β secretion detected 20 h after transfection of 100 nM flagellins in LPS pretreated BMMs. **c** Co-immunoprecipitation assay of flagellin of *E. coli* K12 strain (KF), flagellin of *Salmonella typhi* (SF) and C35 deletion variant of SF with NAIP5 in 293T cells. **d** Co-immunoprecipitation assay of flagellins KF, SF, and C35 replacement variant of SF with NAIP5 in 293T cells. Shown are immunoblots of anti-Flag immunoprecipitates (IP: Flag) and total cell lysates (Input)

We previously showed that flagellins from different bacterial species are differentially recognized by NAIP5; certain flagellins resist detection by NAIP5^14^ and flagellin from *E. coli* K12 (designated as KF for convenience) is 10 times less efficient than flagellin from SF in activating the NAIP5–NLRC4 inflammasome (Fig. 3a, b)^23^. In order to map the region(s) of flagellins that determines recognition by the NAIP5 receptor, a series of KF/SF chimeras and SF truncation were generated. Deletion of the C-terminal 35 amino-acid residues in SF (SF△C35) significantly diminished caspase-1 activation (Fig. 3a) and IL-1β release in bone marrow derived macrophages (Fig. 3b). Biochemically, both KF and SF△C35, contrasting to SF, failed to interact with NAIP5 in the co-immunoprecipitation assay SF (Fig. 3c). The requirement of C-terminal 35 residues in flagellin for NAIP5–NLRC4 inflammasome-mediated detection is highly consistent with that reported in a previous study^22^. Furthermore, replacing the C-terminal 35 residues in SF with those of KF (SF with C35 of KF) disrupted the binding to NAIP5 (Fig. 3d); accordingly, SF with C35 of KF could not efficiently induce the NAIP5–NLRC4 complex formation (Supplementary Figure S4). These data suggest that the C-terminal 35 residues in KF, contrasting to those in SF, cannot support interaction with the NAIP5 receptor.

### Leu-483, Thr-488, and Arg-506 are critical for flagellin recognition by NAIP5

Previous study has identified three conserved leucine residues (3L) in the C-terminal 5 or 5 residues in flagellin^22^, such as L502, L504, and L505 in *Salmonella typhimurium* flagellin (FliC) and L470, L472, and L473 in *Legionella pneumophila* flagellin^14^, that are critical for binding to NAIP5 and activating the inflammasome. The 35 residues including the three leucines are highly conserved in KF. This suggested that the three leucines with the C-terminal 35 amino acids are necessary but not sufficient for efficient flagellin-NAIP5 interaction.

**Fig. 4:**
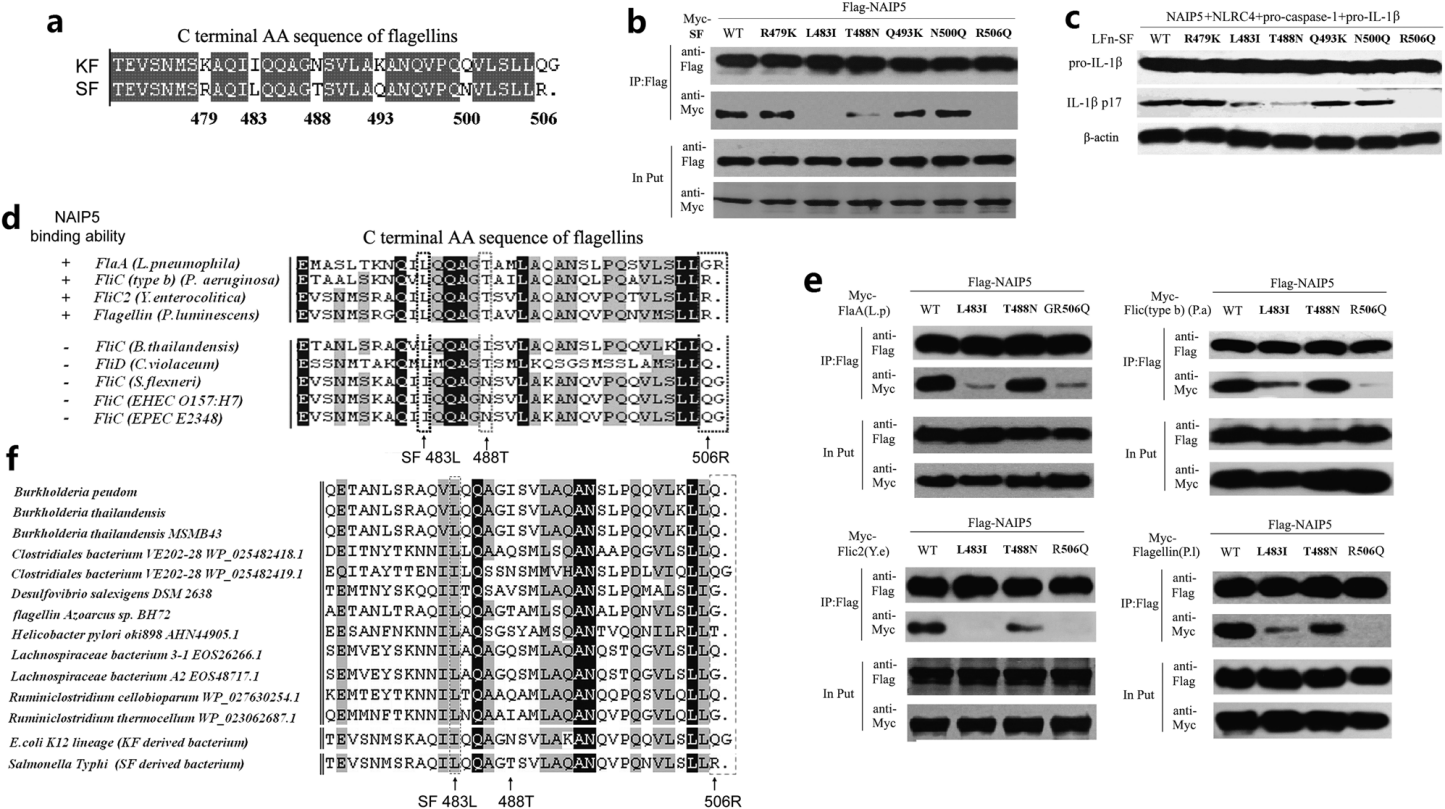
Amino-acid residues 483 leucine, 488 threonine, and 506 arginine are involved in the recognition of flagellins by NAIP5. **a** The alignment of C-terminal 35 amino acids in flagellin of *E. coli* K12 strain (KF) and flagellin of *Salmonella typhi* (SF). **b** Co-immunoprecipitation assays of flagellin SF or its mutants with NAIP5 in 293T cells transfected by plasmid pCS2-Flag-NAIP5 with Myc-flagellin. **c** Reconstitution of flagellin activation of the NLRC4 inflammasome in non-macrophage cells. Lysates from 293T cells transfected with indicated plasmid combinations and stimulated with 5 ng/ml LFn-Flagellins were analyzed for mature IL-1β (p17) by immunoblotting. **d** Alignment of C-terminal sequence in flagellins with or without NAIP5-binding ability according to the yeast two-hybrid experiments^14^. Amino acids critical for the low binding ability of KF with NAIP5 are outlined by wireframes. **e** Co-immunoprecipitation assays of the wild-type flagellins FlaA (L.p), FliC (type b) (P.a), FliC2 (Y.e), and Flagellin (P.l) or their correspondent mutants of SF483, 488, or 506 with NAIP5. **f** Alignment of C-terminal amino-acid sequence of flagellins derived from flagellated commensal bacteria in gut from wild-type C57BL/6 mouse^25^. Expression of transfected inflammasome components for **c** is in Supplementary Figure S5a

To identify additional important residues within the C-terminal 35-residue region, we aligned and carefully compared the sequences of SF and KF. Six amino acids including R479, L483, T488, Q493, N500, and R506 of SF within the 35-residue region were found to be different from those occupying the corresponding position in KF (Fig. 4a). Each of the six residues in SF were then individually mutated into the corresponding residue in KF to obtain SF R479K, L483I, T488N, Q493K, N500Q, and R506Q mutants. In the 293T cell co-immunoprecipitation assay, while SF R479K, Q493K, and N500Q were readily co-precipitated with NAIP5, the L483I, T488N, and R506Q mutants appeared to lose the ability to interact with NAIP5 (Fig. 4b). When delivered into 293T cells expressing NAIP5, NLRC4, pro-caspase-1, and pro-IL-1β by the LFn-fusion strategy, SF L483I and T488N were markedly attenuated and R506Q was nearly completely deficient in triggering IL-1β maturation (Fig. 4c and Supplementary Figure S5a). In contrast, SF R479K, Q493K and N500Q mutants behaved similarly as WT SF in stimulating inflammasome-mediated IL-1β maturation (Fig. 4c). These analyses identify three additional residues Leu-483, Thr-488, and Arg-506 in SF as being critical for NAIP5-mediated innate immune recognition.

In our previous study, four flagellins including those from *L. pneumophila* (FlaA), *Pseudomonas aeruginosa* (FliC (type b)), *Yersinia enterocolitica* (FliC2), and *Photorhabdus luminescens* (Flagellin) are shown to be capable of binding NAIP5, whereas those from EPEC, EHEC, *Shigella flexneri*, *Chromobacterium violaceum*, and *Burkholderia thailandensis* are inert in detection by NAIP5^14^. Consistent with the above observation, all four NAIP5-binding competent flagellins contain the conserved Leu-483, Thr-488, and Arg-506, whereas none of the five NAIP5-binding incompetent flagellins maintain all the three conserved residues (Fig. 4d). Such high-degree correlation supports the important role of the three resides for NAIP5-mediated detection in general. We further generated point mutations of the three residues in the four NAIP5-binding competent flagellins similarly as that with SF. The L483I and R506Q (or GR506Q in the case of FlaA) mutations largely abolished the binding of all four flagellin proteins to NAIP5. The T488N mutation also showed evident disruption of NAIP5 binding in the case of FliC2 from *Y. enterocolitica* and Flagellin from *P. luminescens* but had little effects in *L. pneumophila* FlaA and *P. aeruginosa* FliC (type b) (Fig. 4e), indicating a differential role of this residue for different flagellins.

It has been reported that pathogenic bacteria such as *Salmonella* or *Pseudomonas* species can elicit substantial amounts of mature IL-1β from intestinal mononuclear phagocytes (iMPs) in a NLRC4-dependent manner, but the commensal bacteria *Lactobacillus plantarum*, *Bacterioides fragilis,* or *Enterococcus fecalis* have no such activity^24^. In view of these, we examined a panel of flagellin sequence of mouse gut commensal bacteria and observed a striking phenomenon. None of the twelve commensal-derived flagellins harbored an arginine residue at the extreme C terminus and most of them contained a glutamine similarly as that in KF (Fig. 4f). In addition, isoleucine rather than leucine was also found at the position of residue 483 in certain commensal-derived flagellins^25^ (Fig. 4f).

### The differences in the three amino-acid residues of C-terminal 35 amino acids region make KF unable to activate NAIP5

**Fig. 5:**
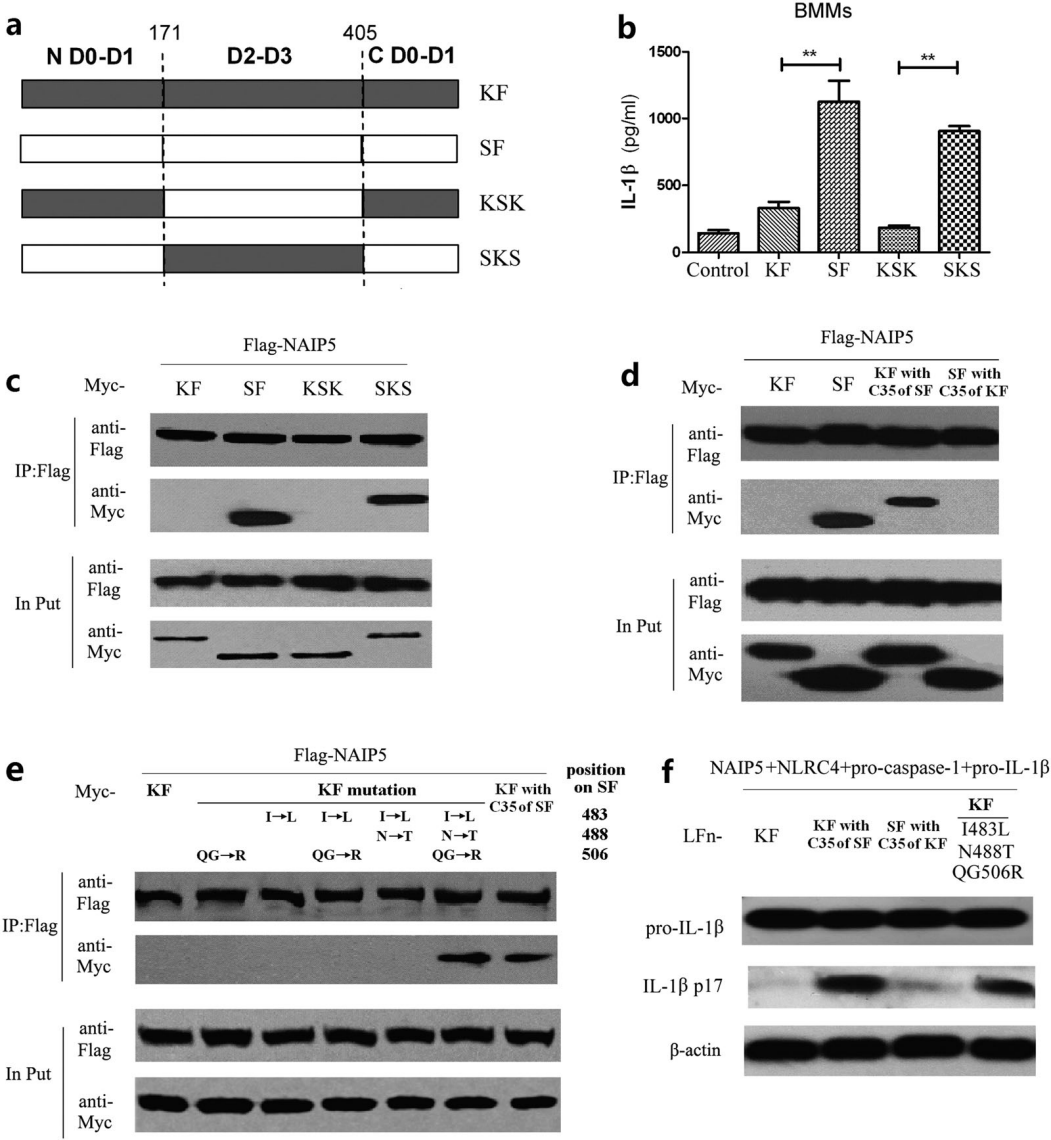
Substitution of amino-acid residues of flagellin of *E. coli* K12 strain (KF) with 483 leucine, 488 threonine, and 506 arginine makes it be recognized by NAIP5. **a** Diagram of flagellin of *E. coli* K12 strain (KF), flagellin of *Salmonella typhi* (SF) and the chimeric flagellins with hyper-variable region substitutions. **b** IL-1β secretion 20 h after transfection with 100 nM flagellins in BMMs. **c** and **d** Co-immunoprecipitation assays of KF, SF, and their chimeric with NAIP5 in 293T cells. **e** Co-immunoprecipitation assays of flagellins KF or its mutations with NAIP5 in 293T cells. **f** Reconstitution of flagellin activation of the NLRC4 inflammasome in non-macrophage cells. Lysates from 293T cells transfected with indicated plasmid combinations and stimulated with LFn-Flagellins were analyzed for mature IL-1β (p17) by immunoblotting. Expression of transfected inflammasome components for **f** is in Supplementary Figure S5b

Given the importance of Leu-483, Thr-488, and Arg-506 for flagellin binding to NAIP5 and the absence of these residues in KF and commensal-derived in flagellins, we then investigated the possibility of recovering the activity of KF by targeted mutation of the three residues. First, we performed domain swapping analyses between SF and KF as illustrated in Fig. 5a. Replacement of the hyper-variable region (D2–D3) in KF with that of SF could recover neither NAIP5–NLRC4 inflammasome-mediated IL-1β secretion in BMMs (Fig. 5b, chimeric KSK) nor the NAIP5-binding ability in the 293T co-immunoprecipitation assay (Fig. 5c, chimeric KSK). Similarly, replacement of the hyper-variable region (D2–D3) in SF with that of KF eliminate neither NAIP5–NLRC4 inflammasome-mediated IL-1β secretion in BMMs (Fig. 5b, chimeric SKS) nor the NAIP5-binding ability in the 293T co-immunoprecipitation assay (Fig. 5c, chimeric SKS). These observations excluded involvement of the hyper-variable region in the differential recognition of KF and SF by NAIP5. In contrast, substitution of the C-terminal 35 residues in KF with those of SF (Fig. 5d, KF with C35 of SF) resulted in the gain of NAIP5-binding activity in the co-immunoprecipitation assay (Fig. 5d, KF with C35 of SF), and ability to stimulate NAIP5–NLRC4 inflammasome reconstituted in 293T cells to produce mature IL-1β (Fig. 5f, KF with C35 of SF). Conversely, SF bearing the C-terminal 35 residues from KF lost both the activity of binding to NAIP5 (Fig. 5d, SF with C35 of KF) and the ability to trigger IL-1β maturation through the reconstituted NAIP5–NLRC4 inflammasome (Fig. 5f, SF with C35 of KF).

To further test whether the loss of Leu-483, Thr-488, and Arg-506 in KF is the cause for its escape from NAIP5-mediated innate immune detection, five KF variants containing single, double, or triple substitutions of Ile-483, Asn-488, and Gln-506 with Leu, Thr, and Arg, respectively, were constructed. As shown in Fig. 5e, the triple mutants, but not the single and double mutants, showed full binding to NAIP5 similarly to that observed with replacing the entire 35-residue region (Fig. 5e, KF with C35 of SF). Consistently, this triple mutant KF could robustly stimulate mature IL-1β production by the reconstituted NAIP5–NLRC4 inflammasome (Fig. 5f and Supplementary Figure S5b). These data suggest that the three-residue variation in KF and likely also other commensal bacteria-derived flagellins is sufficient for their escape from NAIP5 inflammasome receptor-mediated innate immune detection.

## Discussion

In this study, we have first identified critical sequence determinants in NAIP receptor that are important for the specific ligand–receptor binding. Three structural regions, the pre-BIR, BIR1, and HD1 in NAIP2 and NAIP5 are demonstrated to be important for specific pattern-recognition of their respective ligand(s). We further find the C-terminal LRR domain, which is preciously predicted to be the ligand-binding region, is completely dispensable for NAIP2 recognition of the T3SS rod protein, but is required for NAIP5 binding to flagellin. Therefore, the sequence determinants of NAIP2 and five for their respective ligands are completely different though NAIP2 and NAIP5 share very similar domain structures. This finding highlights the complexity of the differential sequence determinants of NAIPs for their respective ligand specificity.

For NAIP2, our data indicate that three amino acids Asp-18, Arg-108, and Arg-667 in pre-BIR, BIR1, and HD1, respectively, are critical for binding to the T3SS inner rod protein. This demonstrates for the first time that the pre-BIR and BIR1 confer specificity of NAIP2 for its specific interaction with rod protein. This finding sheds light on the function of the BIRs in NAIPs, which suggests that pre-BIR and BIR1 are likely directly involved in the binding of specific bacteria ligand. The single-point mutant R108S of NAIP2 being defective in binding to BsaK in the yeast two-hybrid analyses and abolishing the oligomerization of NLRC4 indicated that the BIR1 domain plays important role in specific pattern-recognition of rod protein and subsequent assembly of the NAIP–NLRC4 complex. Our data also suggest that the pre-BIR domain with the Asp-18, which is critical in both ligand binding and assembly of the NAIP–NLRC4 complex, also has an important role as BIR1 and HD1 for NAIP2 to recognize the rod protein specifically. The analysis of domain swapping and progressive truncations of the C-terminal portion of NAIP2 not only identifies that the HD1 in NAIP2 dictates its ligand specificity for rod protein recognition, but also rules out the involvement of the other two domains WHD and HD2 in central NBD-associated domains in NAIP2 for its ligand specificity. This is not consistent with the conclusion obtained by Tenthorey and colleagues, in which HD1, WHD, HD2, and part of the unannotated domain of NAIP2 were all shown involved in dictating specific recognition of PrgJ^21^. The discrepancy might be partly explained by the different systems used. We used the yeast two-hybrid system and 293T co-precipitation system to directly test the regions involved in the specific recognition of rod protein BsaK. But Tenthorey et al. used NLRC4 inflammasome reconstitution system and detected the formation of NLRC4 complex and the NLRC4 inflammasome activation for evaluation of PrgJ recognition^21^. To finally resolve the discrepancy, more intensive study and precise structure data are needed.

For NAIP5, we unfold a different scenario from NAIP2 for the specific ligand recognition. Our data indicate that NAIP5, different from NAIP2, requires the LRR domain to bind to flagellin and activate NAIP5–NLRC4 inflammasome. Tenthorey et al. previously found that LRR domains of NLRs are not necessarily pathogen-detection domains by generating and analyzing a set of reciprocal NAIP2/5/6 chimeras, in which the N-terminal domains were fused to the C-terminal domains^21^. Consistent with their result, our data show LRR region of NAIP2 participates neither in the ligand recognition nor function as an autoinhibitory domain, but in the NAIP–NLRC4 oligomer formation. However, our analyses with three chimera NAIP5/2 molecules and progressive truncations of NAIP5 in LRR region from the C terminus demonstrate that the LRR domain is required for NAIP5 to bind to flagellin and activate NAIP5–NLRC4 inflammasome. The different structural domains required for NAIP2 and NAIP5 to bind their respective ligands may result from the different size between flagellin and the rod protein (BsaK in this study), in which the smaller rod protein BsaK can be readily recognized by N-terminal BIR domains and NBD-associated domains of NAIP2. On the other hand, referring to the model of the wheel-like structure^8, 21^, it is hard for the small size rod protein to interact with both ends of the NAIP protein, whereas this is feasible for the bigger size protein flagellin. It is intriguing to speculate that the NAIP5 also needs its LRR domain to form more stable complex with flagellin to initiate assembly of the wheel-like flagellin-NAIP5–NLRC4 complex vice versa. Our study suggests that the auto-inhibited NLRC4 has certain plasticity to be triggered by different NAIP-ligand complex, in which the “open form” specific NAIP-ligand complex^21^ has activity to interact with the auto-inhibited NLRC4 and initiate subsequent cascade of self-propagation of NLRC4. It seems that the C-terminal LRR domain has differential roles in different NAIPs, which might function both as an autoinhibitory domain^26–28^ and a “sensor” for NAIP5 to recognize flagellin, but only as an autoinhibitory domain for NIAP2. This phenomenon suggests that different NAIP forms specific pattern by combination of different molecular domains to achieve their specific pattern-recognition ability. Further investigation on the mechanism of specific ligand-NAIP–NLRC4 inflammasome activation is needed.

The present study has also identified critical sequence determinants at the ligand side that are essential for ligand–receptor interaction and being recognized specifically. We further identified three non-conserved amino-acid residues, Leu-483, Thr-488 and Arg-506 in the conserved C35 amino acids region of flagellin of *Salmonella typhi* (SF), being critical in the binding of SF with NAIP5. The three amino-acid residues confer flagellin being differentially recognized by NAIP5. The three residues corresponding Leu-483, Thr-488, and Arg-506 in SF are mostly conserved in a set of NAIP5-binding competent flagellins, whereas the three different corresponding amino-acid residues Ile-483, Asn-488, and Glu-506/Gly-507 in from flagellin of *E. coli* K12 strain (KF) are usually located in a set of flagellins derived from flagellated commensal bacteria in healthy mouse gut. The correlation of the three amino acids with whether the flagellin is susceptible to NAIP5-mediated immune detection gives us hint that bacteria might avoid NAIP5 detection by mutation at these three amino-acid sites in flagellin. Three more bacteria examples are found in flagellins derived from *S. flexneri*^29^, *EPEC,*^14^ and *EHEC O157:H7*^14^, which cannot be detected by NAIP5. The sequences of C35 amino-acid residues in all the three bacteria flagellins are identical with that in flagellin of *E. coli* K12 strain (KF) by alignment of sequence (Supplementary Figure S6). It is suggested that the mutation in the C35 region is likely a mechanism engaged by some severe pathogenic bacteria for evading the detection and clearance by NAIP5–NLRC4 inflammasome. Furthermore, we find from the ligand side that at the extreme C terminus, the amino-acid residue Arg is in none of the commensal-derived flagellins. Most of them contain a Gln similarly as that in KF. In addition, Ile rather than Leu is also present at the position of residue 483 in certain commensal bacteria-derived flagellins (Fig. 4f). Our data suggest that the absence of Leu-483, and Arg-506, which makes flagellins incapable to interact with NAIP5, is likely an important strategy for commensal bacteria being null to be recognized by NAIP5 and avoidable of unnecessary NAIP5–NLRC4 pathway activation. Theoretically, host should distinguish commensal bacteria that provide benefits to host from virulent pathogenic bacteria^30–32^. Franchi and colleagues reported that the commensal bacteria in the gut can be distinguished from pathogenic bacteria by iMPs through evasion of NAIP–NLRC4 inflammasome recognition^24^. Our finding provides some molecular basis of this distinguishable recognition of flagellin by NAIP5 from ligand side. In other words, we find amino-acid determinants in flagellin for NAIP5 to determine whether or not to recognize and respond to bacterial flagellin.

In summary, our study demonstrates that the sequence determinants for NAIPs are complex and the interaction between one NAIP and its cognate ligand is determined by their specific sequences. However, little is known yet about how the limited NAIPs deal with the vast diversity of ligands different in size and composition from different pathogenic as well as commensal bacteria strains and species. More systemic studies from both receptor and ligand in the future are needed to elucidate the mechanisms of specific and differential pattern-recognition.

## Material and Methods

### Plasmids, antibodies, and reagents

DNAs for flagellin were amplified from the corresponding bacterial genomic DNA. BsaK and PrgJ DNAs were amplified from *B. thailandensis* E264 and *S. typhimuriumLT2* strains, respectively. PA expression plasmid was obtained from Addgene. Expression plasmids for pro-caspase-1 and pro-IL-1β were provided by X. Wang (University of Texas Southwestern Medical Center). NAIP2, NAIP5 and NLRC4 were amplified as described previously^14^. For mammalian expression, cDNAs for all NLR proteins were cloned into modified pCS2 vectors with an N-terminal Myc, HA, or Flag epitope tag. All chimeras, truncations and point mutations were generated by standard molecular biology procedures. All plasmids were verified by DNA sequencing.

Antibodies for caspase-1 and Myc epitopes were obtained from Santa Cruz Biotechnology. Other antibodies used in this study include IL-1β (3ZD; Biological Resources Branch, National Cancer Institute), HA epitope (Covance) and Flag M2 (Sigma). 293T cells obtained from ATCC were grown in Dulbecco’s modified Eagle’s medium containing 10% fetal bovine serum and 2 mM l-glutamine at 37 °C in a 5% CO_2_ incubator. Cell culture products were from Invitrogen and all other chemicals were Sigma-Aldrich products unless noted.

### Yeast two-hybrid and co-immunoprecipitation assays

For Yeast two-hybrid assay, indicated flagellin and BsaK genes were cloned into the bait vector pLexAde, and mouse Naip2, Naip5, Naip chimeras, truncations, or point mutations cDNAs were cloned into the prey vector pVP16. The bait and prey plasmids were co-transformed into the reporter *Saccharomyces cerevisiae* strain L40 by using the lithium acetate method. Two-hybrid assays were performed by following a classical procedure^33^.

For immunoprecipitation, 293T cells were transfected with indicated plasmids. Cells were harvested and lysed in a buffer containing 50 mM Tris-HCl (pH 7.6), 150 mM NaCl, and 1% Triton X-100 supplemented with a protease inhibitor mixture (Roche Molecular Biochemicals). Precleared lysates were subjected to anti-Flag M2 immunoprecipitation by following the manufacturer’s instructions. The beads were washed three times with the lysis buffer and the immunoprecipitates were eluted in the SDS sample buffer followed by immunoblotting analysis. All the immunoprecipitation assays were performed more than three times and representative results are shown in the figures.

### Purification of recombinant proteins

Recombinant flagellins was cloned into pET28a vector (Addgene) for recombinant expression in *E. coli* BL21 (DE3) and purified as described previously^23, 34^. In brief, recombinant flagellins were prepared and purified by affinity chromatography on a Ni-NTA column (Qiagen) and dialyzed with phosphate-buffered saline (PBS) at 4 °C. Endotoxin contaminants was removed as previously described^23^. The purified proteins were treated with Acrodisc syringe filters (Pall, Port Washington, NY, USA) and preserved in −80 °C. Quantification of the purified proteins was performed using the Bradford assay. Residual content was determined by Limulus assay (Associates of Cape Cod). Endotoxin values of recombinant proteins for immunization were < 0.005 EU/μg. RAW 264.7 cells, which could sensitively respond to lipopolysaccharides (LPS) and bacteria DNA but not flagellin^35, 36^ were used further to exclude the presence of residual bacteria DNA and LPS contaminations.

Recombinant LFn-flagellin or BsaK were cloned into pET28a-LFn vector (Addgene) for recombinant expression in *E. coli* BL21 (DE3) as described previously^14, 37, 38^. In brief, bacteria were harvested and lysed in a buffer containing 50 mM Tris-HCl (pH 7.6), 300 mM NaCl, and 25 mM imidazole. His-tagged proteins were purified by affinity chromatography using Ni-NTA beads (Qiagen). To remove the majority of endotoxin contaminants, proteins bound onto the Ni-NTA column were subjected to an additional wash with 60% isopropanol in the wash buffer (30 column volumes). Proteins were then eluted with 250 mM imidazole in 50 m M Tris-HCl (pH 7.6) and 300 mM NaCl. Eluted samples were further dialyzed against a buffer containing 50 mM Tris-HCl (pH 7.6) and 150 mM NaCl to remove the imidazole. Protein concentrations were estimated by Coomassie blue staining of sodium dodecyl sulfate polyacrylamide gel electrophoresis (SDS–PAGE) gels using BSA as the standards.

### NLRC4 inflammasome reconstitution in 293T cells

For reconstitution in 293T, cells were seeded into a six-well plate 12 h before transfection with indicated combinations of plasmids using the Vigofect reagents (Vigorous). The amounts of plasmids used are 2 µg for pro-human IL-1β, 50 ng for caspase-1, 100 ng for NLRC4 and 100 ng for NAIP proteins. Twenty-four hours later, final concentration of 0.5 µg/ml LFn-flagellin or 1 µg/ml LFn-BsaK together with 1 µg/ml PA proteins was added into the culture medium for another 12 h if not specially indicated. Cells were harvested and lysed in a buffer containing 50 mM Tris-HCl (pH 7.6), 150 mM NaCl, and 1% Triton X-100. Lysates were resolved onto SDS–PAGE gels followed by anti-IL-1β immunoblotting analysis. All the reconstitution experiments were performed more than three times and representative results are shown in the figures.

### Assay for the oligomeric NAIP–NLRC4 inflammasome complex formation

293T cells were seeded into a six-well plate 12 h before transfection with plasmids encoding Flag-NLRC4, HA-NAIPs, or NAIP mutants and Myc-FliC/PrgJ using Vigofect Reagent (Vigorous Inc.). 24 h later, transfected cells were washed with PBS for three times and lysed in the native sample buffer. The lysates were then subjected to blue native PAGE analysis (Invitrogen), as previously described^24^, to analyze the oligomerization of NLRC4 and also the presence of NAIPs in the NLRC4 inflammasome complex.

### Flagellin transfection-mediated inflammasome activation assays in macrophages

Female C57BL/6 mice at 8–10 weeks of age were obtained from Beijing Laboratory Animal Research Center and kept in the Animal Center of Wuhan Institute of Virology (WIV), Chinese Academy of Sciences (CAS) under specific pathogen-free conditions. Animal studies were performed according to Regulations for the Administration of Affairs Concerning Experimental Animals in China (1988), the Guideline for Animal Care and Use, WIV, CAS. BMMs were prepared from the femurs of mice by culture in RPMI 1640 containing 10 % FBS with the addition of recombinant mouse macrophage colony stimulating factor (M-CSF, 25 ng/ml, eBioscience) and non-essential amino acids for 7 days. For cytokine and cell death assays, BMMs were seeded at the density of 1 × 10^5^ cells/well in 96-well plates, pretreated with LPS at a concentration of 50 ng/ml for 3-h and transfected with flagellins using DOTAP (Roche Diagnostics) as previously described^23^. Supernatants were collected 20 h after transfection. For the LDH release assay, cell lysis buffer was added 1 h before the collection of the supernatant samples. The release of lactate dehydrogenase (LDH) was measured by the Cytotoxicity Detection Kit^PLUS^ (LDH) (Roche). The cytokine concentrations were measured using ELISA kits (mouse IL-1β, eBioscience). To exclude the release of pro-IL-1β from dead cells, the IL-1β values shown were normalized to the release of pro-IL-1β from lysed macrophages (mature IL-1β = total IL-1β signal – pro-IL-1β lysis × percent release of LDH) as previously described^2^.

The method of caspase-1 activation analysis was as described previously^23^. For brief: 8 × 10^5^ BMMs seeded into 12-well plates were pretreated with LPS and then transfected with flagellins. In some cultures, the endogenous caspase-1 was blocked with the specific inhibitor z-YVAD-fmk (Calbiochem) at a final concentration of 2–8 μM. Supernatants were collect and cells were lysed in the presence of a protease inhibitor cocktail (Pierce). After centrifugation, cytoplasmic lysates were combined with the supernatants. Proteins were precipitated with 10 % trichloroacetic acid, washed with ethanol, and subjected to SDS gel electrophoresis and Western blotting analysis with anti-caspase-1 p10 (Santa Cruz Biotechnology), anti-β actin (Sigma).

### Statistical analysis

All of the results are presented as the means + standard error of the mean from triplicates of one experiment that repeated at least three times and were analyzed by means of nonparametric two-tailed Student's *t*-tests using GraphPad Prism 5. Statistical significance is indicated by * (*P* < 0.05), ** (*P* < 0.01), and *** (*P* < 0.001). *A P* value < 0.05 was considered statistically significant.

## Electronic supplementary material

Supplementary Figures and Figure legends
