# Supplementary material for: Sequence determinants of specific pattern-recognition of bacterial ligands by the NAIP–NLRC4 inflammasome
Source: Cell Discov. 2018 May 8;4:22. doi: 10.1038/s41421-018-0018-1 (PMC5938239; doi:10.1038/s41421-018-0018-1)
Supplement: Supplementary file 1 — Supplementary Figures and Figure legends [file 41421_2018_18_MOESM1_ESM.pdf]

Supplementary Information

Supplementary Figure legends

Supplementary Figure S1 Expression of transfected inflammasome components in 293T cell reconstitution assays of the oligomeric NAIP-NLRC4 inflammasome complex or NLRC4 inflammasome activation. Replicate transfection for each of the reconstitutions shown in Fig. 1c (a), Fig. 1e (b), Fig. 1g (c) and Fig. 1h (d) was subjected to anti-Myc immunoprecipitation followed by anti-Myc or anti-HA immunoprecipitation followed by anti-HA immunoblotting.

Supplementary Figure S2 C-terminal region from WHD to LRR domain in NAIP2 is dispensable for binding to the TTSS rod proteins BsaK and EscI. Co-immunoprecipitation assays of rod proteins BsaK, EscI with NAIP2, NAIP5, NAIP2/5 chimeras indicated in Fig. 1a and NAIP2 truncation NAIP2T3 in 293T cells. Shown are immunoblots of anti-Flag immunoprecipitates (IP: Flag) and total cell lysates (Input).

Supplementary Figure S3 Expression of transfected inflammasome components in 293T cell reconstitution assays of NLRC4 inflammasome activation. Replicate transfection for each of the reconstitutions shown in Fig. 2f was subjected to anti-Myc immunoprecipitation followed by anti-Myc or anti-HA immunoprecipitation followed by anti-HA immunoblotting.

Supplement Figure S4 The C35 region of SF is important of flagellin induced NAIP5/NLRC4 complex formation. Co-immunoprecipitation assays of flagellins with NAIP5 with the presence of NLRC4. 293T cells were transfected by plasmid pCS2-Flag-NAIP5 with Myc-flagellin or pCS2- Flag-NLRC4 with HA-NAIP5 and

Myc-flagellin for 30 hours. Shown are immunoblots of anti-Flag immunoprecipitates (IP: Flag) and total cell lysates (Input).

Supplementary Figure S5 Expression of transfected inflammasome components in 293T cell reconstitution assays of NLRC4 inflammasome activation. Pro-caspase-1, NLRC4 and NAIP5 proteins were tagged with an N-terminal Myc and HA epitopes respectively. replicate transfection for each of the reconstitutions shown in Fig. 4c (a) and Fig. 5f (b) was subjected to anti-Myc immunoprecipitation followed by anti-Myc or anti-HA immunoprecipitation followed by anti-HA immunoblotting.

Supplement Figure S6 Some flagellins without NAIP5 binding ability possess the same C terminal 35 amino acids with KF. The C terminal region of KF was aligned with flagellins of *S. flexneri, EHEC O157:H7* and *EPEC E2438*.

Fig.S1

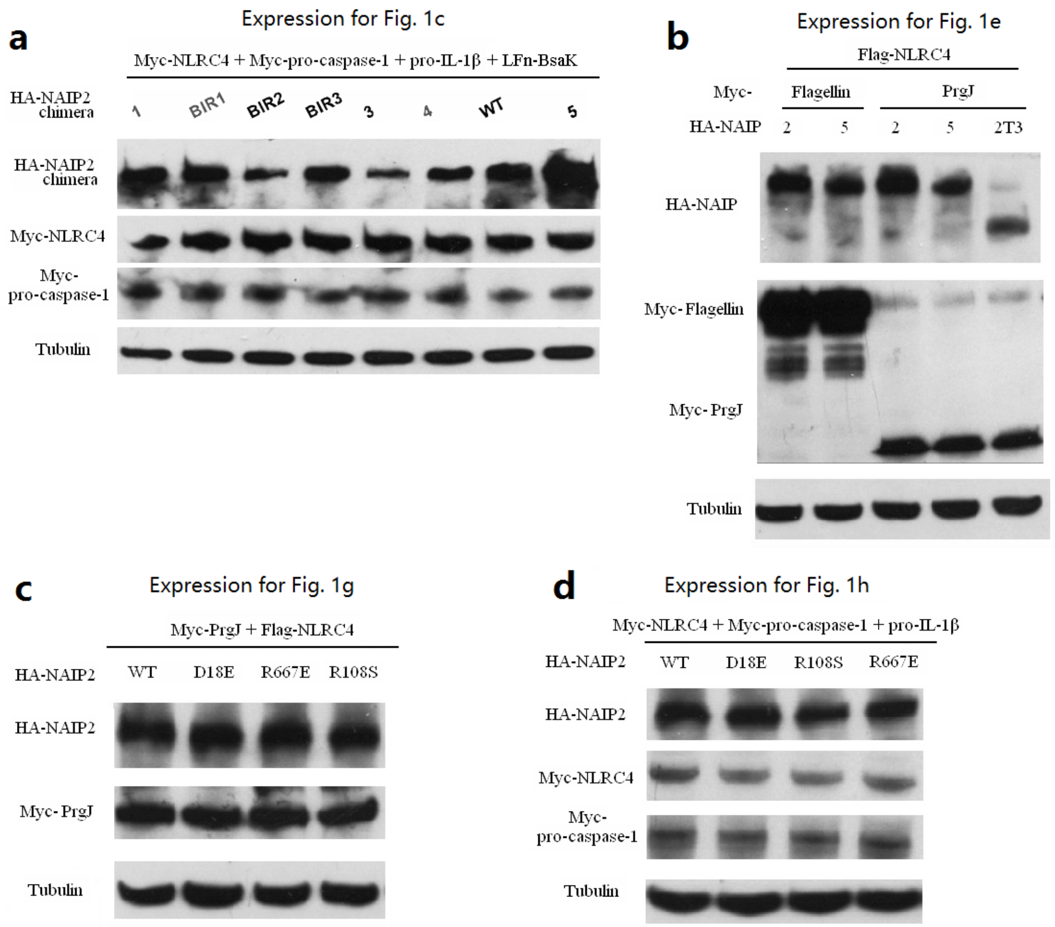
a
Expression for Fig. 1c
Myc-NLRC4 + Myc-pro-caspase-1 + pro-IL-1β + LFn-BsaK
HA-NAIP2 chimera
1
BIR1
BIR2
BIR3
3
4
WT
5
HA-NAIP2 chimera
Myc-NLRC4
Myc-pro-caspase-1
Tubulin
b
Expression for Fig. 1e
Flag-NLRC4
Myc-
Flagellin
PrgJ
HA-NAIP
2
5
2
5
2T3
HA-NAIP
Myc-Flagellin
Myc-PrgJ
Tubulin
c
Expression for Fig. 1g
Myc-PrgJ + Flag-NLRC4
HA-NAIP2
WT
D18E
R667E
R108S
HA-NAIP2
Myc-PrgJ
Tubulin
d
Expression for Fig. 1h
Myc-NLRC4 + Myc-pro-caspase-1 + pro-IL-1β
HA-NAIP2
WT
D18E
R108S
R667E
HA-NAIP2
Myc-NLRC4
Myc-pro-caspase-1
Tubulin

Fig.S2

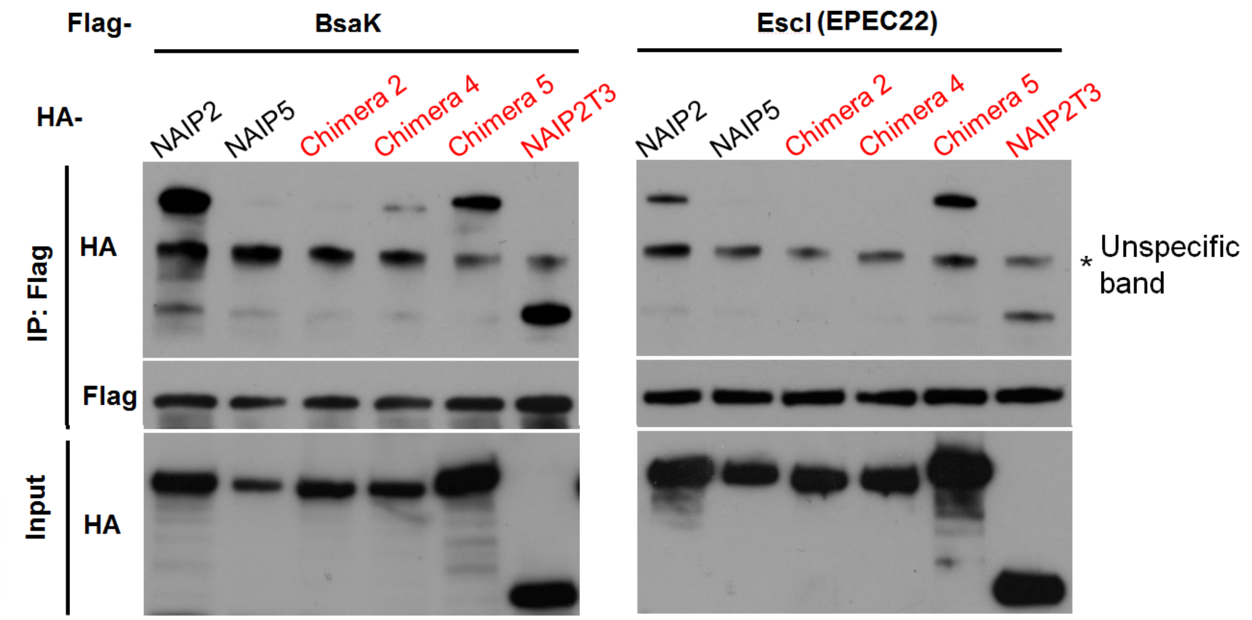

Flag-
BsaK
EscI (EPEC22)
HA-
NAIP2
NAIP5
Chimera 2
Chimera 4
Chimera 5
NAIP2T3
NAIP2
NAIP5
Chimera 2
Chimera 4
Chimera 5
NAIP2T3
IP: Flag
HA
Flag
Input
HA
* Unspecific band

Fig. S3

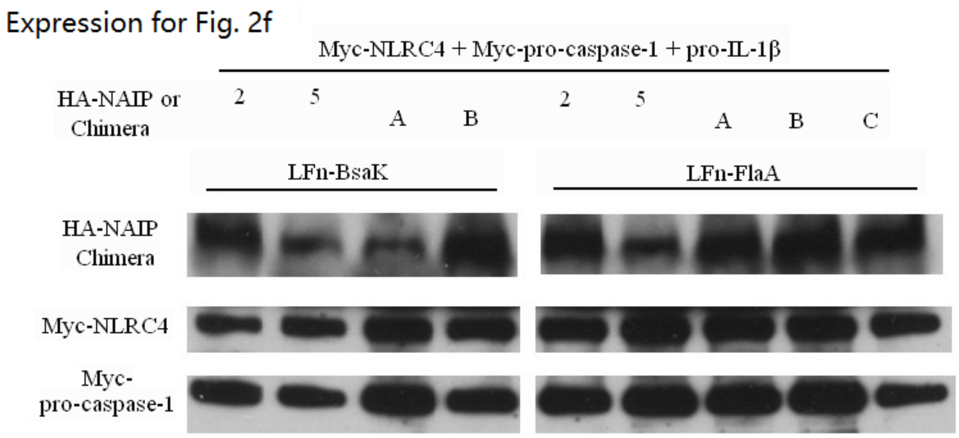
Expression for Fig. 2f
Myc-NLRC4 + Myc-pro-caspase-1 + pro-IL-1β
HA-NAIP or Chimera
2
5
A
B
2
5
A
B
C
LFn-BsaK
LFn-FlaA
HA-NAIP Chimera
Myc-NLRC4
Myc-pro-caspase-1

Fig. S4

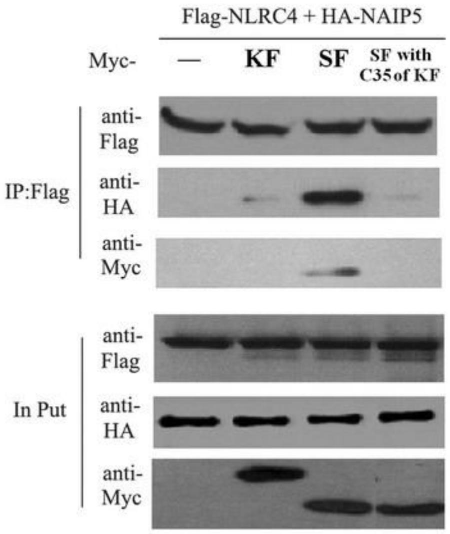
Flag-NLRC4 + HA-NAIP5
Myc-
—
KF
SF
SF with C35 of KF
IP:Flag
anti-Flag
anti-HA
anti-Myc
In Put
anti-Flag
anti-HA
anti-Myc

Fig.S5

Expression for Fig. 4c

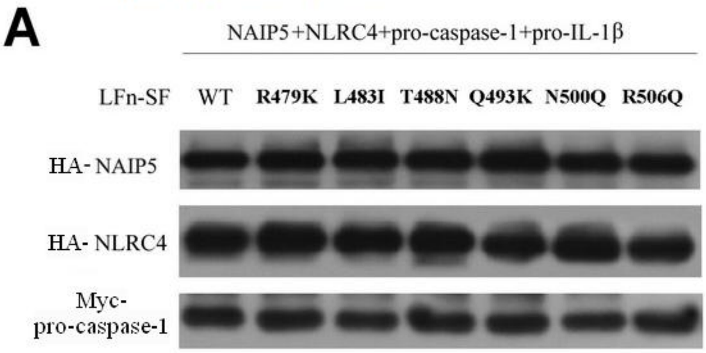

Expression for Fig. 5f

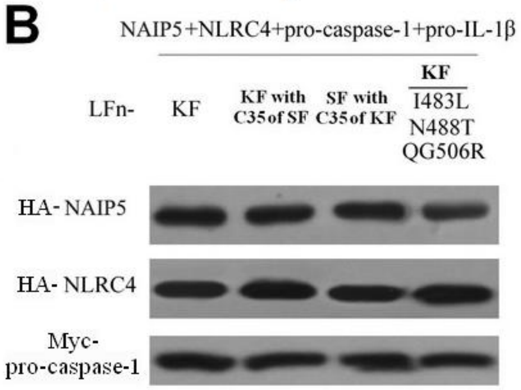

Fig. S6

```
KF          NLSEAQSRIQDADYATEVSNMSKAQIIQQAGNSVLAKANQVPQQVLSLLQG
EHEC O157   NLSEAQSRIQDADYATEVSNMSKAQIIQQAGNSVLAKANQVPQQVLSLLQG
EPEC E2348  NLSEAQSRIQDADYATEVSNMSKAQIIQQAGNSVLAKANQVPQQVLSLLQG
S.flexneri  NLSEAQSRIQDADYATEVSNMSKAQIIQQAGNSVLAKANQVPQQVLSLLQG
```
